# Pro- and Antiangiogenic Factors in Gliomas: Implications for Novel Therapeutic Possibilities

**DOI:** 10.3390/ijms22116126

**Published:** 2021-06-07

**Authors:** Magdalena Groblewska, Barbara Mroczko

**Affiliations:** 1 Department of Biochemical Diagnostics, University Hospital in Białystok, 15-269 Białystok, Poland; magdalena.groblewska@umb.edu.pl; 2 Department of Neurodegeneration Diagnostics, Medical University of Białystok, 15-269 Białystok, Poland

**Keywords:** angiogenesis, vasculogenesis, angiogenic switch, endothelial cells, glioma, central nervous system tumor, blood-brain-barrier, tumor-related brain edema, chemotherapy

## Abstract

Angiogenesis, a complex, multistep process of forming new blood vessels, plays crucial role in normal development, embryogenesis, and wound healing. Malignant tumors characterized by increased proliferation also require new vasculature to provide an adequate supply of oxygen and nutrients for developing tumor. Gliomas are among the most frequent primary tumors of the central nervous system (CNS), characterized by increased new vessel formation. The processes of neoangiogenesis, necessary for glioma development, are mediated by numerous growth factors, cytokines, chemokines and other proteins. In contrast to other solid tumors, some biological conditions, such as the blood–brain barrier and the unique interplay between immune microenvironment and tumor, represent significant challenges in glioma therapy. Therefore, the objective of the study was to present the role of various proangiogenic factors in glioma angiogenesis as well as the differences between normal and tumoral angiogenesis. Another goal was to present novel therapeutic options in oncology approaches. We performed a thorough search via the PubMed database. In this paper we describe various proangiogenic factors in glioma vasculature development. The presented paper also reviews various antiangiogenic factors necessary in maintaining equilibrium between pro- and antiangiogenic processes. Furthermore, we present some novel possibilities of antiangiogenic therapy in this type of tumors.

## 1. Introduction

Malignant glioma is among the most common types of primary tumors of the central nervous system (CNS) [1]. The increased formation of new blood vessels is one of the characteristic features of this group of malignancies. The pathophysiological processes of glioma angiogenesis play key roles in the development of these tumors and their growth, already from the earliest stages [2,3]. Angiogenesis and invasion in gliomas are related with the production of a multitude of growth factors, cytokines, chemokines and other proteins [4].

The aim of this literature study was to present various aspects of neoangiogenesis in gliomas. Moreover, the current review discusses the role of various proangiogenic factors in driving glioma progression, as well as various antiangiogenic factors, which are necessary in maintaining an equilibrium between pro- and antiangiogenic processes. In addition, we discuss the new therapeutic possibilities in the treatment of this type of tumor.

## 2. Physiological Vasculo- and Angiogenesis

### 2.1. Vasculogenesis

Angiogenesis is defined as a formation of new blood vessels from already existing vasculature [5], which is especially important in embryogenesis, and in many physiological processes in adult organisms, such as the female menstruation cycle and pregnancy [6,7], wound healing [8] and the formation of granulation tissue [9]. Angiogenesis is also observed in various nonmalignant pathologies, for instance ischemic diseases, diabetic retinopathy, or autoimmunological disorders [5].

Physiological angiogenesis is preceded by vasculogenesis, a generation of the first embryonic vessels and the development of the circulatory system. These vessels originate from mesoderm cell precursors (hemangioblasts) of the yolk sac, which subsequently differentiate in situ into endothelial cell progenitors (angioblasts), and, finally, endothelial cells (EC) [10]. It was demonstrated that migration and differentiation of angioblasts occurs in the response to local stimuli, such as cytokines, especially growth factors, and certain chemokines as well as some components of the extracellular matrix (ECM) to form new blood vessels [11]. 

### 2.2. Angiogenesis

The process of angiogenesis is thought to be the most responsible for the growth of blood vessels [11]. During angiogenesis, the development of previously formed vasculature is continued by two essential mechanisms: endothelial sprouting and intussusceptive microvascular growth (IMG), which is the splitting of vessels previously arisen in vasculogenesis. The sprouting mechanism is based on endothelial cell migration, proliferation and tube formation. The process begins from the budding of a monolayer of endothelial cells to form capillaries, followed by the extending of the vascular trees. Angiogenetic sprouting is initiated by tissue hypoxia in the regions that lack blood vessels. Deficiency of vasculature leads to the shortage of nutrients and oxygen within tissue. This results in the secretion of vascular endothelial growth factors (VEGF) by parenchymal cells [12]. The next step of vasculature evolution is the merging of growing vascular sprouts, which may create anastomoses. These processes are regulated by various proangiogenic growth factors synthesized by hypoxic cells [13]. 

Intussusceptive angiogenesis is an alternative mechanism of blood vessel formation, where an existing blood vessel is split into two new ones [14]. IMG has several steps, when existing vessel lumens are divided by formation and insertion into the vessel lumen columns of interstitial tissue and specific tissue folds. This type of vessel formation is especially important in embryonic development. The reorganization of existing cells during IMG allows for an enormous increase in the number of capillaries, without a corresponding increase in the number of endothelial cells [15]. Moreover, IMG also includes the formation of loops in situ in the wall of large veins [16]. 

The walls of developing blood vessels are composed of two distinct cell types: endothelial cells, which form internal layer and mural cells, forming the outer layers of the vascular wall. The mural cells may be subdivided into pericytes, embedded within the basement membrane of capillaries [17], and vascular smooth muscle cells (vSMCs), associated with arteries and veins [18]. Pericytes share their basal membrane with the endothelial cells and are generally considered as contractile cells, which participate in the regulation of blood flow in the microcirculation [19,20].

## 3. Proangiogenic Factors

In physiological conditions, processes of angiogenesis are highly controlled by various factors—cytokines, chemokines, and growth factors, which may have pro- or antiangiogenic properties. Uncontrolled and deregulated reactivation of angiogenesis is also a crucial step of the progression of various malignant tumors, including gliomas. The most important stimulators of these mechanisms are the members of VEGF family and their receptors, molecules from fibroblast growth factor (FGF) family, angiopoietin/Tie system, transforming growth factor-beta (TGF-β), metalloproteinases (MMPs), and chemokines. 

### 3.1. VEGF 

As it was mentioned above, tissue hypoxia triggers the synthesis and secretion of VEGFs, the essential regulators of angiogenesis and vascular permeability. The VEGF family includes several members: VEGF-A, VEGF-B, VEGF-C, VEGF-D and placental growth factor (PlGF). Among them, the most important factor in blood vessels forming is VEGF-A [21]. Alternative splicing of VEGF-A generates two subfamilies with specific functions: proangiogenic, termed VEGF_xxx_, or antiangiogenic proteins, named VEGF_xxx_b [22]. VEGF-A is a potent proangiogenic growth factor, synthesized by endothelial cells, smooth muscle cells of blood vessels, and fibroblasts. Moreover, other cells, such as keratinocytes, macrophages and monocytes [23], mast cells, eosinophils and T lymphocytes, as well as cancer cells have also the ability to produce this cytokine [24]. 

The activity of VEGF-A is concerned mainly with vascular endothelial cells. VEGF-A has been shown to stimulate the mitogenesis in endothelial cells and their migration in vitro [25]. However, this growth factor may have effects on the functions of many other cell types, including neurons, kidney epithelial cells or cancer cells. VEGF-A may also act as a chemoattractant for the migration of monocytes/macrophages, contributing to macrophage recruitment and M2 polarization [26]. It also may increase MMPs activity, which facilitates the creation of blood vessel lumina. 

### 3.2. FGF Family

Proteins from fibroblast growth factor family are another group of promoters of angiogenesis [27,28,29]. Among them, the most recognized proteins are FGF1, also termed acidic fibroblast growth factor (aFGF), and FGF2, named as basic fibroblast growth factor (bFGF), which are more potent angiogenic factors than VEGF-A. FGFs bind to four high affinity tyrosine kinase receptors (FGFRs 1–4), out of which FGFR1 and -2 may be expressed on the surface of endothelial cells [30]. Binding FGFs to specific receptors and activation of signal transduction cascade results in the induction of a strong angiogenic response on endothelium of blood vessels, chemotaxis and proliferation of endothelial cells and their migration [30,31]. While FGF-1 induces the proliferation and differentiation of all cell types necessary for building an arterial vessel, including smooth muscle cells, FGF-2 promotes the proliferation of endothelial cells and their organization into tubelike structures [31]. 

It was suggested that the contribution of FGFs to the signaling of angiogenesis is rather indirect and requires activation of the VEGF system [28]. FGFR1 (fms-related tyrosine kinase-2) also may play an indirect role in vasculogenesis [32]. FGF signaling induces VEGF expression and influences other growth factors, such as PDGF and certain chemokines. This activity of FGF contributes to the development of mature vessels and collateral arteries, by enhancing the degradation of ECM and upregulation of proteolytic enzymes, such as urokinase-type plasminogen activator (uPA) and MMPs in endothelial cells [33]. 

### 3.3. Angiopoietins and Angiopoietin-Like Proteins

Angiopoietins are also important regulators of angiogenesis, involved with controlling microvascular permeability, endothelial barrier function, vasodilation and vasoconstriction [34] as well as vessel sprouting and endothelial cell migration [35]. Angiopoietins are the ligands for the tyrosine kinase receptors with immunoglobulin-like and EGF-like domain 1 (Tie-1) and the endothelial-specific receptor tyrosine kinase (Tie-2 or TEK), expressed in endothelial cells [36]. 

Until now, there are four angiopoietins identified: Ang-1, Ang-2, Ang-3 (mice orthologue), and Ang-4 (human) [37]. Among them, the most recognized are Ang-1 and Ang-2. Ang-1 is a key regulator of vessel stabilization, which reduces vascular permeability and protects the adult vasculature against plasma leakage during inflammation [35,38]. The opposite properties may be ascribed to Ang-2, which inhibits biding of Ang-1 to Tie2, which results in vessel destabilization and disruption of angiogenesis in vivo [39]. Moreover, Ang-2 sensitizes endothelial cells to proinflammatory cytokines, such as TNF-alpha [40], and may promote neovascularization in collaboration with VEGF [34].

Among various angiogenic factors, there is also a group of structurally similar molecules, termed “angiopoietin-like proteins” (ANGPTLs), which do not interact with classical receptors of angiopoietins Tie-1 and Tie-2 [41,42,43]. ANGPTL proteins have various properties and are involved in a diversity of functions, including angiogenesis, proliferation of endothelial cells, metabolism of lipoproteins, regulation of hematopoiesis and apoptosis. Among them, ANGPTL2 is the most proangiogenic activity regulator [41]. 

### 3.4. TGF-β Superfamily

The transforming growth factor superfamily is a large group of cytokines, including various signaling proteins, such as TGFs, bone morphogenetic proteins (BMPs) or activins [44]. TGF-β is the prototypic member of this cytokine family and is a potent stimulator of angiogenesis. There are three TGF-β isoforms known—TGF-β1, TGF-β2, and TGF-β3 [45]. TGF-β ligands are produced by a variety of normal and malignant cells, including osteoblasts, platelets, and all leukocytic cell lineages [46], as well as placenta chorionic cells, megakaryocytes, cardiac myocytes, chondrocytes, renal distal tubules, adrenal cortex, and ovarian glandular cells [47]. In the sites of wound healing, this cytokine is also expressed abundantly by epithelial cells, associated fibroblasts and myofibroblasts, as well as by infiltrating immune cells, such as macrophages and T lymphocytes [48]. 

TGF-β binds to heteromeric complexes of serine/threonine kinase receptors I and II [49]. There are seven mammalian type I receptors, also named as activin receptor-like kinases (ALK1-7) and five type II receptors [50,51]. TGF-β signaling by various receptors depends on context, and can either promote or suppress endothelial migration, proliferation, permeability, and sprouting. TGF-β signaling may be also driven via betaglycan, the type III TGF-β co-receptor (TβRIII), which may play important roles as a regulator of cell migration, invasion, cell growth, and angiogenesis [52,53]. Disturbed signaling pathways of TGF-β may be linked to various diseases, including autoimmune and cardiovascular diseases, fibrosis, and cancer. Moreover, loss of betaglycan from tumor cells is frequently associated with increased cell migration and invasion, increased angiogenesis, and increased tumor progression [53]. 

TGF-β modulates various pro- and antiangiogenic factors affecting endothelial and mural cells [44]. Moreover, genetic mutations resulting in knock-out of TGF-β superfamily proteins or their receptors and loss of their expression are related with various defects in vasculo- and angiogenesis. Lack or deficits of TGF-β signaling pathways, including deletion of TGF-β1, as well as knock-out of receptors resulted in the development of impaired, leaky, and hyper-dilated blood vessels in animal models of angiogenesis [54]. Multiple effects TGF-β1 on vascular endothelial cells are related to VEGF and VEGF receptor-2 (VEGFR-2), which mediate TGF-β1-induced apoptosis [55]. 

### 3.5. MMPs

Angiogenesis, especially the migration and invasion of the endothelial cells, would be impossible without the degradation of components of ECM, basement membranes of existing blood vessels, and local tissue structures [56,57]. The remodeling of the ECM allows these cells to invade into the surrounding stroma and enables the sprouting of vascular network. In these processes, proteolytic enzymes called MMPs are the crucial players [58]. This family of zinc-containing endopeptidases includes 28 members [59]. The expression of at least 14 of them can be found in the vascular endothelium [60]. According to the substrate specificities of proteolytic activity and their common structural domain architecture, MMPs may be classified into at least six subfamilies: collagenases, gelatinases, stromelysins, matrilysins, membrane-type MMPs (MT-MMPs), and other MMPs [61]. The subgroup of stromelysins includes MMP-3, -10, and -11, the enzymes with a broad substrate specificity, which may degrade many ECM proteins, such as proteoglycans, fibronectin, and laminin [56,62]. MMP-1, -8, and -13 from the collagenases subfamily are also enzymes associated with angiogenesis and their loss leads to the permanent disruption of the ECM [63]. The subgroup of gelatinases includes MMP-2 and MMP-9, the proteinases with the ability to degrade types IV, V, VII, and X native collagens, fibronectin and laminin [62,64]. 

MMPs have multiple effects on angiogenesis, not only by degrading ECM components [62]. MMPs are also involved in the disruption of tight junctions between pericytes and endothelial cells, promoting proangiogenic endothelial cell proliferation. Some of these enzymes may also enhance angiogenesis by helping pericytes to detach from vessels experiencing angiogenesis or by cleaving endothelial cell-cell adhesions [65]. Furthermore, the degradation of ECM results in the release and activation of angiogenic growth factors bound in the ECM, such as TGF-β, FGF and VEGF, and in the generation of pro-migratory fragments of various ECM components [66,67,68,69,70]. Moreover, activity of MMPs may cause the presenting of some proangiogenic integrin binding sites which are normally obscured within cryptic the ECM [71]. 

Proteolytic activity of MMPs is controlled by tissue inhibitors of metalloproteinases (TIMP-1, TIMP-2, TIMP-3, and TIMP-4), proteins which also play a crucial role in angiogenesis regulation by inhibiting neovascularization [63]. Activation of MMPs may be prompted by certain proangiogenic factors, such as VEGF, bFGF, and angiogenin. Similarly, proangiogenic TNF-α or chemokine C-X-C motif ligand 8 (CXCL8) have the ability to stimulate the production of MMP-2, -8, and -9 in endothelial cells, thus controlling the angiogenesis process [72]. 

### 3.6. Proangiogenic Chemokines and Their Receptors

Chemokines constitute another group of factors important for angiogenic processes. They play a role as the stimulators of the directional migration of various types of cells, especially various subclasses of leukocytes, towards a chemokine gradient [73]. Chemokines are synthesized by various types of cells, including neutrophils, monocytes, macrophages, T lymphocytes, and fibroblasts, as well as neural, endothelial and epithelial cells [74]. The chemokine superfamily accounts for over 50 ligands, which share a conserved protein structure within N-terminal domain, with two cysteine residues [75]. Depending on their molecular structure, members of chemokine family may be divided into four sub-families, CXC with single amino-acid (AA) separating the cysteine tandem, CC with residues not separated by any other AA, XC with a single N-terminal cysteine, and CX_3_C with three AA between these two cysteine residues [76]. Chemokine signaling also includes circa 25 chemokine receptors (CKRs), named according to the predominant type of chemokine they bind [77]. CKRs also share a conservative structure with 50% homology within the same class and 30% between different classes [78]. 

Chemokines are important factors controlling homeostasis and the immune system. Although chemotaxis was the first originally described function of chemokines [79], they also play an important roles in angiogenesis. Among homeostatic chemokines, CXCL12 is the most primitive and conservative molecule, considered as necessary for life. CXCL12, together with its receptor CXCR4, creates a specific signaling pathway, called “CXCL12-CXCR4 axis”, which is particularly important for the development of many organs, including the heart and vascular system [77,80]. CXCL12/CXCR4 axis is central for the vascularization of various organs [81,82,83]. One of the most important inflammatory chemokines is CXCL8, also known as interleukin 8 (IL-8), which creates together with receptors CXCR1 and CXCR2 another specific chemokine-receptor signaling pathway called “CXCL8-CXCR1/2 axis”. 

Activity of chemokines as mediators of angiogenesis is one of the essential functions of these proteins [84]. Their importance as the regulators of angiogenesis was demonstrated especially in CXC chemokines [85,86], which may be further divided into two subfamilies, depending on the presence of “ELR motif” in the N-terminal domain of chemokine molecule, which is a conserved sequence of the three amino acids Glu-Leu-Arg [87]. By specific receptor binding, the presence of ELR motif defines the chemotactic properties of CXC chemokines and their angiogenic potential [84,88]. CXCL8 is the prototypic proangiogenic CXC ELR (+) chemokine, which promotes endothelial cell migration, invasion, and proliferation [89]. All human CXC ELR (+) chemokines may bind to the chemokine receptor CXCR2, which was identified in human microvascular endothelial cells. Moreover, the blockade of this receptor by neutralizing antibodies resulted in the lack of angiogenic activity induced by CXC ELR (+) chemokines [90]. Furthermore, CXC ELR (+) chemokines can also signal through chemokine receptor CXCR1 [91]. CXC ELR (+) chemokines mediate angiogenesis by the recruitment of proangiogenic hematopoietic and immune cells as well as endothelial progenitor cells to the neovascular niche [92]. They also activate chemokine receptors on endothelial cells, which results in the stimulation of their chemotaxis and tubular morphogenesis. Chemokines may also stimulate angiogenesis via direct interaction between chemokine/chemokine receptor complexes and receptor tyrosine kinase receptors as well as with other proangiogenic cytokines, such as VEGF and FGF [85]. 

Another large group of proangiogenic factors is the CC subfamily. CC chemokines are chemoattractants for various cells, including lymphocytes, eosinophils, basophils, and monocytes, as well as dendritic and natural killer (NK) cells [79]. Although they are involved mainly in the inflammatory response, immunological surveillance, and lymphocyte homing, these chemokines play also the role of proangiogenic factors [86,93,94,95]. One CC chemokine with proangiogenic activity is CCL2, which may stimulate angiogenesis directly, through its receptor CCR2, expressed on endothelial cells [96]. In response to CCL2, these cells exhibit chemotaxis and formation of endothelial tube in vitro [97]. Moreover, it was demonstrated that CCL2 significantly increases surface expression of MT1-MMP, its clustering, and activity in human endothelial cells [98]. These results indicate that CCL2-induced tube formation is highly dependent on MT-1 MMP activity. Another proangiogenic CC chemokine is CCL11, a chemoattractant for eosinophils. This chemokine may directly induce chemotaxis and proliferation of endothelial cells by binding to CCR3 receptor [93]. It was demonstrated that CCL11 induced microvessel sprouting in a rodent model of angiogenesis, and these effects were greater and observed earlier than after stimulation with VEGF [99]. This chemokine may have also indirect proangiogenic activity, resulting in the formation of blood vessel in vivo. It was observed in mice that stimulation by CCL11 resulted in the infiltration of eosinophils, which consequently released other proangiogenic factors including TGF-β [93]. CCL16 is also an important factor in angiogenesis [94], activating an angiogenic program in vascular endothelial cells by binding to CCR1, which induces migration of endothelial cells in a dose-dependent manner, differentiation of endothelium into capillary-like structures and formation of endothelial tube in vitro. Moreover, CCL16 cooperates with VEGF and other proangiogenic signaling pathways through increased basal production of CXCL8 and CCL2 [94]. 

The only known member of the CX_3_C chemokine family, CX_3_CL1, is a chemoattractant for monocytes, NK cells, and lymphocytes. It was revealed that recombinant CX_3_CL1 may induce the proliferation, migration, and formation of endothelial tube in vitro as well as stimulate the angiogenesis in vivo [100]. CX_3_CL1 and its receptor, CX_3_CR1, are expressed on endothelial cells [100,101]. The CX_3_CL1-CX_3_CR1 axis is necessary for microvessel budding, their maturation, and vascular structural integrity [102]. It was revealed that monocytes expressing this receptor may be stimulated by CX_3_CL1 to differentiate into the smooth muscle-like cells during healing of blood vessel walls after their injury [102]. On the contrary, disturbed activity of the CX_3_CL1–CX_3_CR1 axis resulted in the growth of smaller, poorly developed, leaky, and hemorrhagic microvessels in experimental models of neovascularization [103]. CX_3_CL1-induced angiogenesis is associated with phosphorylation of some enzymes, such as endothelial nitric oxide (NO) synthase (eNOS), especially in hypoxic conditions. During ischemia, eNOS stimulates the synthesis of NO, which directly promotes angiogenic processes by controlling the expression of other proangiogenic factors such as VEGF, FGFs and angiopoietins, as well as genes involved in extracellular matrix metabolism, including MMPs [104]. 

Summary of proangiogenic factors in normal angiogenesis is presented in Table 1.

## 4. Endogenous Inhibitors of Angiogenesis

Angiogenesis is also controlled by some naturally occurring endogenous inhibitors, which display a broad spectrum of biological activity and may influence various angiogenic mechanisms and processes: downregulation of genes expressed in endothelial cells, interference with the formation and migration of these cells, or inhibition of the endothelial tube morphogenesis.

### 4.1. Angiostatin

Angiostatin is a polypeptide of approximately 200 amino acids, which is produced by the autoproteolytic cleavage of plasminogen, or by MMPs’ enzymatic activity [105]. Although the exact mechanisms of angiogenesis inhibition by angiostatin are not known, it seems that this protein may induce apoptosis of endothelial cells and inhibit their proliferation and migration [106,107]. Moreover, it was suggested that the primary antiangiogenic mechanism of angiostatin is the inhibition of MMP-dependent endothelial cell migration [108]. It was also proposed that angiostatin decreases transiently the phosphorylation of mitogen-activated protein kinases ERK-1 and ERK-2 and diminishes activation of these enzymes by proangiogenic growth factors bFGF and VEGF [107]. 

### 4.2. Endostatin

Another naturally occurring inhibitor of angiogenesis, endostatin, is a 20-kDa derivative of type XVIII collagen, from its C-terminal globular domain [109]. Endostatin is a broad-spectrum antiangiogenic factor. This protein has the ability to downregulate 12% of genes used by human endothelial cells, which are involved in cell cycle control and apoptosis [110]. In addition, endostatin has also an antimigratory influence on endothelial cells via the suppression of c-myc expression, mainly in microvascular endothelial cells, which may result in cell death [110]. 

Endostatin may also inhibit the proliferation of endothelial cells and their organization into new blood vessels, which may suppress growth of malignant tumors, both primary and metastatic ones [109]. Suppression of angiogenesis by endostatin also includes an interference with the proangiogenic activity of growth factors such as bFGF and VEGF [111]. The interference of endostatin with FGF signaling disrupts the reorganization of cytoskeleton and adhesion between endothelial cells and ECM [112], which also results in the regulation of endothelial cell apoptosis.

### 4.3. Vascular Endothelial Growth Inhibitor

Vascular endothelial growth inhibitor (VEGI), also known as TNF-like ligand 1A (TL1A) or TNF superfamily member 15 (TNFSF15), is an antiangiogenic factor abundantly expressed in endothelial cells [113]. VEGI signals through two receptors, death receptor 3 (DR-3), for which is a sole ligand, and decoy receptor 3 (DcR-3), a soluble protein from TNFR superfamily. VEGI may inhibit angiogenesis both in vitro and in vivo, acting as an autocrine factor inducing apoptosis in endothelial cells and arresting their proliferation [114].

### 4.4. Decoy Receptors

A protein which can recognize specific growth factors or cytokines and bind them, but has no signaling ability is called as “decoy receptor”. These proteins are believed to be inhibitors in various signaling pathways. Receptor 1 for VEGF (VEGFR-1), named also FLT-1, is expressed by endothelial cells and acts a high affinity receptor for VEGF-A, VEGF-B, and placenta growth factor [115]. Although the role of VEGFR-1 in angiogenesis is not known precisely, its deletion results in abnormal overgrowth of endothelial cells and early embryonic lethality [116]. The soluble form of VEGFR-1 plays a role as a decoy receptor, which may negatively modulate angiogenesis [117]. This decoy characteristic of VEGFR-1 is required for normal development and angiogenesis. By sequestering and trapping VEGF, VEGFR-1 inhibits the activity of VEGFR-2, thus preventing VEGFR-2 from binding to VEGF [117,118].

Another decoy receptor which inhibits angiogenesis is neuropilin (NRP1). NRP1 is a membrane-bound coreceptor to FLT-1, a tyrosine kinase receptor for VEGF isoforms, VEGF_165_ [119]. NRP1 plays also important roles in axon guidance [120], as well as in cell survival, migration, and invasion [121]. NRP1 modulates VEGF binding and bioactivity, thus regulating VEGF-induced angiogenesis [119,122]. The expression of NRP1 on endothelial cells increases the affinity of VEGF_165_ for the receptor VEGFR-2, leading to enhanced chemotaxis and mitogenesis of endothelial cells [122]. On the contrary, the soluble form of neuropilin (sNRP1) has the opposite effect of membrane bound NRP1 and has anti-VEGF activity [123]. It was demonstrated that sNRP1 is secreted by cells as a 90-kDa protein that binds VEGF_165_ and inhibits its binding to endothelial cells and VEGF_165_-induced tyrosine phosphorylation of kinase domain receptor (KDR) in endothelial cells. It was also demonstrated in vivo that injections of sNRP-1 could inhibit the progression of acute myeloid leukemia in mice, which may indicate that sNRP1 inhibits tumor angiogenesis depending on VEGF [123].

### 4.5. Antiangiogenic Chemokines and Chemokine Receptors

Apart from chemokines with proangiogenic activity, there is also a group of antiangiogenic factors within this superfamily of proteins. CXC chemokines lacking ELR motif, or “CXC ELR (−) chemokines” are mostly chemoattractive for lymphocytes T and NK cells [88]. Members of this chemokine group bind mainly to the chemokine receptor CXCR3, which is expressed predominantly on T lymphocytes, and some B cells and NK cells, although it may be also present on the endothelial cells [124]. It was demonstrated that CXCR3 may mediate the angiostatic activity of ELR (−) chemokines [125,126]. CXCR3 ligands, CXCL4, CXCL9, CXCL10, and CXCL11 chemokines, may directly stop chemotaxis of endothelial cells and prevent the formation of endothelial tube induced by growth factors. The inhibition of angiogenesis may be also mediated by angiostatic chemokines through a positive feedback loop, in which CXC ELR (−) chemokines stimulate the recruitment of NK and Th1 cells [127]. Moreover, these chemokines induced the regression of newly formed cords in vitro and the loss of blood vessels in vivo. Antiangiogenic ligands of CXCR3 could inhibit proliferation and migration of human microvascular endothelial cells [86].

Another angiostatic activity was observed for CXCL10 chemokine, which could induce dissociation of newly formed vessels and their regression during wound healing, as well as endothelial cell death [128]. On the contrary, neutralizing antibodies against this chemokine and its receptor CXCR3 inhibited dissociation of vascular cord mediated by CXCL10 [128]. Inhibition of angiogenesis may be also related with binding of angiostatic chemokines to the receptors expressed on endothelial cells, which may induce apoptosis or regression of vessels. Antiangiogenic activity of ELR (−) may occur also by binding and trapping of proangiogenic growth factors, which results in inhibition of their activity.

Endogenous inhibitors of angiogenesis are summarized in Table 2.

## 5. Gliomas—Malignant Brain Tumors: Anatomical, Biological and Clinical Considerations

It is estimated that primary tumors of CNS may represent approximately 2% of all cancer cases worldwide [129]. However, these tumors belong to the leading causes of cancer-related death [130]. The reason of such unfortunate outcome may be that incidence rates of primary CNS tumors is similar to mortality rates of these malignancies [130]. Primary CNS tumors constitute a highly heterogeneous group of neoplasms and include tumors of the brain, meninges, cranial nerves or spinal cord. CNS tumors have also various frequencies of specific histological types within different age groups and variegated course of the disease.

According to the 2016 World Health Organization (WHO) classification of tumors of the central nervous system, these neoplasms may be classified as grade I, II, III, or IV [131], and may represent low- or high-grade tumors. Among all malignant tumors of CNS, the most frequent are gliomas, which account over 40% of all primary CNS malignancies. Gliomas are the tumors of neuroepithelial origin, derived from oligodendrocytes and astrocytes, the cellular components of the glia. They arise in the glial tissue and primarily occur in the brain.

Low-grade infiltrating gliomas (LGG) are assigned as WHO grade II tumors and include astrocytomas, oligodendrogliomas, and oligoastrocytomas. They often develop in young, otherwise healthy patients. These tumors generally seem to have better survival time in comparison with high-grade gliomas (HGG). Although grade II LGGs are characterized with relatively less invasive course of the disease, they also have a significant potential to progress to more aggressive tumors. It was reported that about 50% of patients diagnosed with low-grade gliomas may progress into high-grade gliomas within 5 years. The progress of LGG into their malignant high-grade counterparts may occur within 5 years after its gross-total resection (<1 cm residual tumor) even in about 50% of patients [132,133].

More aggressive CNS tumors, such as anaplastic astrocytoma, mixed anaplastic oligoastrocytoma and anaplastic oligodendroglioma represent grade III glial tumors, but glioblastoma multiforme (GBM), a WHO grade IV tumor, is characterized with the worst prognosis among all CNS tumors. It is estimated that high-grade anaplastic astrocytomas and GBM constitute about 55% of all neuroepithelial tumors in adults [134].

Among glioblastoma multiforme tumors, there are two different types: primary and secondary. Majority of all GBM tumors (approximately 90%) occur as primary GBM (PrGBM), the tumors developing de novo, without a pre-existing lower-grade glioma. Typically, they progress in older patients, over 60 years of age. On the contrary, secondary GBM tumors (ScGBM) arise from preexisting tumors, especially from grade II or III astrocytomas or mixed gliomas bearing certain genetic mutations [131]. ScGBM tumors typically occur in younger patients (before 45 years of age) and represent about 10% of cases.

Primary and secondary GBM have a similarly unfavorable outcome, with a median survival shorter than one year after diagnosis. PrGBM and ScGBM also have similar histology, although these tumors exhibit distinct genetic alterations, such as mutations in isocitrate dehydrogenase (IDH), *TP53* or *PTEN* mutations, as well as 19q and 22q loss of heterozygosity (LOH) [135,136,137]. Therefore, these tumors may be considered as two different diseases [138].

The precise etiology of GBM is still unclear, although several genetic alterations have been reported as factors affecting the development of CNS tumors, including gliomas [131,139]. These alterations include mutations, activation of oncogenes, loss of telomerase and induction of aneuploidy, as well as epigenetic alterations and molecular changes. Furthermore, the regulatory role over certain steps of gliomagenesis may be ascribed to some cytokines, chemokines, and their receptors. These steps include tumor proliferation, evasion of tumor cells from immunosurveillance, the transition from low to high-grade gliomas as well as tumor angiogenesis.

## 6. Neoangiogenesis in Glioma Development

An exuberant and anomalous vasculature is one of the defining histological characteristics of GBM. The pathological diagnosis of glioblastoma was led by the presence of microvascular proliferation within high-grade astrocytic neoplasm, accompanied by tumor necrosis [140]. The way in which CNS solid tumors such as GBM meet the increasing demand for oxygen and nutrients by generating an increased blood supply is a very complex and multistep process. These mechanisms include described above vasculogenesis, angiogenesis, and vascular co-option, as well as vascular mimicry (VM) and glioblastoma-endothelial cell transdifferentiation, closely related with VM. Importantly, there are extensive interactions and interlinks between these processes, with potential overlapping among them.

It is believed that angiogenesis is the principal method of vessel formation in gliomas. There are three distinct steps discriminated in this process: a breakdown of blood vessels followed by the degradation of their basement membrane and surrounding ECM, which results in the migration of endothelial cells and the formation of new blood vessels [140]. After regression of existing vessels and breakdown of the basement membrane, endothelial cells proliferate and migrate toward tumor cells expressing proangiogenic molecules [141]. The invasion of endothelial cells represents an integral part of the angiogenic process.

The process of the formation of new blood vessels in tumors is called “neoangiogenesis”, to emphasize the fact that tumor angiogenesis differs significantly from physiological angiogenesis. Similarly to normal tissues, gliomas (and other neoplastic tumors) also use blood vessels for the delivery of oxygen and nutrients, necessary for their growth. As the tumor develops, less vascularized regions are formed within its growing mass. They are characterized with the imbalance between the supply and demand of oxygen [142]. Hypoxia occurring in the developing tumor is a physiological stimulus, which induces the expression of certain proangiogenic genes. Low oxygen state leads also to an increased synthesis of described above proangiogenic mediators, such as VEGF, PDGF, MMPs, or angiopoietins, as well as hypoxia-inducible factor 1 alpha (HIF-1α), a major controlling factor in glioma invasiveness and angiogenesis [142,143]. Moreover, hypoxia results in the alterations in angiogenic signaling pathways within glioma stem cells. The process of neoangiogenesis is complex and involves multiple players.

### 6.1. Angiogenic Switch

The changes in cell signaling initiate the beginning of neoangiogenesis, named the “angiogenic switch”. Angiogenic switch is a process of the development of angiogenic phenotype by tumor cells, which results in a rapid formation of new blood vessels [144]. Furthermore, angiogenic switch promotes tumor growth and invasiveness, through activation of oncogenes and upregulation of the expression/signaling of angiogenic pathways, as well as by downregulation of tumor-suppressor genes [142,145,146]. Overexpression of proangiogenic forms of VEGFs may be related with the development and growth of various malignant tumors, including gliomas. While solid tumors cannot grow beyond a limited size without an adequate blood supply, expression of VEGF gives them the ability of growth and metastasizing. Endothelial cells expressing receptors for VEGF are the target cells for the angiogenic switch triggered by VEGF-A [147,148].

Moreover, the angiogenic switch is also associated with the activity of MMPs, especially gelatinases. MMP-2 and MMP-9 may contribute to the angiogenic switching in pre-malignant tumors [149]. It was demonstrated that these enzymes are highly expressed in WHO grade III brain tumors [150]. MMP-2 and MMP-9 have a synergistic effect on endothelial basement membrane degradation in gliomas and mediate the release of ECM-sequestered VEGF [151]. Activity of MMPs is also related with abnormal interactions between endothelial cells and pericytes, different than in normal development of blood vessels. Glioma blood vessels show also increased endothelial cell proliferation, which is a key feature of HGG tumors [152,153,154]. An imbalance between pro- and antiangiogenic signals within the tumor microenvironment results in the secretion of MMPs by pericytes and their detachment from the basement membrane [155]. Another effect of MMPs activity is that endothelial cells loosen their adherence and tight junctions (TJs), and migrate into ECM, allowing for leakage of plasma proteins outside blood vessels [155].

Malignant glioma growth and progression are characterized by remarkable, characteristic increase in the angiogenesis. However, as there are multiple phenotypes of gliomas, the tumors characterized with great heterogeneity, there is also marked diversity in the tumor vasculature and multiple phenotypes of the neoangiogenic processes. Moreover, the angiogenic activity of these tumors does not have to correlate with tumor aggressiveness, although in some types of neoplasms it may be a prognostic factor [156]. An example of such an angiogenic switch is observed during the aforementioned transformation of some LGGs into infiltrative malignant HGGs. When LGGs are rather characterized by next to no neovascularization, the abundant hypervascularization is specific for HGGs, including high-grade astrocytoma, oligodendrogliomas, and ependymomas [157]. Such hypervascularization, measured as vessel density, offers the glioma a blood supply sufficient for its exponential growth. Maximal vessel density is observed in GBM, which belongs to the most vascularized tumors [153]. The explanation might be that low-grade CNS tumors undergo angiogenic switch, which may allow quick progression and malignant transformation toward high-grade tumor [158,159]. HGGs express some transcriptional alterations in angiogenesis-associated factors, such as VEGF, FGF, and epidermal growth factor (EGF), which correlate with neovascularization in human GBM samples [160], and the upregulation of these genes may also play a role in activating the angiogenic switch.

### 6.2. Aberrant Blood Vessel Structure

Growth of gliomas and colonization within the brain tissue is associated with the development of highly aberrant and poorly functional blood vessels [152,161]. These new blood vessels are characterized by disorganized architecture and tend to be tortuous and leaky, with increased permeability. This may result in vasogenic edema in the vicinity of the tumor [162]. These vessels also often exhibit delayed maturation. Moreover, a chaotic and irregular blood flow within tumor additionally contributes to intratumoral hypoxia, which might create a positive bio-feedback loop increasing neoangiogenesis [163]. What is interesting is that primary and secondary HGG glioblastomas exhibit distinct blood vessel morphology and different angiogenic phenotypes [164]. While a predominant expression of VEGF-A was observed in PrGBMs, a significantly higher expression levels of PDGF were detected in ScGBMs. This suggests that these tumors may evolve through different genetic pathways, including VEGF and PDGF [164].

The atypical features of the tumor vasculature described above, which differ them from resting blood vessels, may be the result of imbalance between the expression of various pro- and antiangiogenic factors, such as cytokines and chemokines, as well as the status of tumor suppressor protein p53, which can regulate key angiogenic cytokines and inhibitors. Cellular expression of these factors may vary between different tumor types [156]. Additionally, neovascularization of tumor may occur using a number of diverse biological mechanisms, which may vary between tumor type and its anatomic location. This imbalance between pro- and antiangiogenic factors, both their levels and activity, may arise from the interactions between the tumor cells and the infiltrating inflammatory cells, such as tumor-infiltrating macrophages (TAMs) [165].

### 6.3. Mechanisms of Tumor Vessel Development

Glioma neoangiogenesis may be processed by rerouting or remodeling existing vascular system. It also can occupy some mechanisms observed in normal angiogenesis, such as sprouting new branches from pre-existing vessels and proliferation of endothelial cells from local vessels. Interestingly, these different processes may occur simultaneously within the same tumor tissue. Moreover, the tumor vasculature may develop by joining of pre-existing vessels in the process of colonization of circulating cells, mainly endothelial, from the bone marrow (BM).

However, these processes may also involve participation of nonendothelial cells, such as progenitors or cancer stem cells. This extension of the vasculature facilitated by bone-marrow-derived cells (BMDCs) is observed especially in malignant tissues [166], but may occur also in ischemic normal tissues [167,168]. It was shown that trafficking of BMDCs is regulated by hypoxic gradients through HIF-1 induction of CXCL12 [169,170]. BMDCs are also responsible for generating a microenvironment for tumor growth, invasion, and metastasis [170,171].

### 6.4. Vascular Mimicry

Vascular mimicry is the ability of tumor cells to form functional vessel-like networks, which is an alternative mechanism of neovascularization, employed by various malignant tumors [172], in which a blood supply for their growth and hematogenic propagation may be provided. This process results in the de novo generation of some microvascular channels by aggressive, metastatic and genetically deregulated tumor cells [173]. These structures form a kind of vascular-patterned networks, which imitate normal host endothelial blood vessels. VM was first described in highly aggressive cutaneous melanoma tissues, as vascular channels devoid of endothelial cells, which contained red blood cells inside [174]. What is more, the cells lining these channels strongly expressed angiogenic genes.

The result of VM is the disorganized and destabilized structure of such new pseudo-vessels, their high permeability and tortuous shape, as well as abnormal endothelial and pericyte coverage [175,176]. Although VM was originally demonstrated in human melanoma models, other types of tumors, including glioma, may also use this mechanism for their progression. In another study, conducted on GBM model, similar vascular channels showed tumor cells linings, which maintained morphological characteristics of glioma [177]. Immunohistochemical staining of this tumor with typical glioma markers, CD34 and CD31, confirmed that these channels were not lined by endothelial cells.

Even if endothelial cells do not participate in VM, the tumor cells obtain an endothelial phenotype, which is called “glioblastoma-endothelial cell transdifferentiation”. This process allows tumor to create some vascular structures containing plasma and blood cells, which are embedded in ECM. Interestingly, it was suggested that hypoxia and HIF-1 play roles in promoting the transdifferentiation of GBM cells [178]. An endothelial-like cells derived from tumor were colocalized with hypoxic portions of the GBM tumor, while reduced oxygen concentration enhanced these morphological changes.

### 6.5. Disturbed Blood–Brain Barrier

Glioma neoangiogenesis leads not only to the formation of leaky and permeable blood vessels characterized by disorganized architecture, but also influences the blood-brain barrier (BBB) [179]. The main function of normal BBB is the regulation of molecular and cellular transport across the brain endothelium and the maintaining of water and ion homeostasis in the CNS [180]. This barrier consists of numerous neurovascular units (NVU), specialized structures acting as “gatekeepers” within the CNS. The function of NVUs is controlling of transcellular and paracellular passage of various molecules and cells [181,182].

NVUs are composed of brain capillary endothelial cells, which are covered by a specific form of ECM, the basal lamina. Endothelial cells forming the walls of brain capillaries are connected by tight junctions, which prevent paracellular transport of small and large water-soluble compounds from the circulation to the brain [183]. Outside this basal membrane, endothelium is surrounded by pericytes and astrocytic endfeet [184]. Within NVUs are present also other types of brain tissue cells, such as interneurons and perivascular microglia, which come into contact with endothelial cells, pericytes and astrocytes. NVUs are very closely integrated into the brain neuropil, with extremely small perivascular space, which results in low permeability of brain capillaries [185]. Therefore, various specific transporters are necessary for providing the compounds essential for the brain energy metabolism and clearance [186]. NVUs also create loose interconnections with neuroparenchymal cells, neuronal endings and microglia [187].

Although barrier genesis, the formation of the BBB, begins early in embryonal development, its final maturation occurs in the postnatal period and includes the appearance of intercellular TJs and a reduction of transcytosis and fenestrations in endothelial cells [188,189]. In the mature brain, the astrocytes send their endfeet towards the perivascular basal lamina and form the astroglial layer. Ensheathment of brain capillaries by astrocytic endfeet provides a complete covering of the brain microvessels [190]. These developmental processes are influenced by pericytes, astrocytes and neurons [191].

The functioning of the normal, mature BBB is under the strict control of astrocytes [192,193]. Astrocytes regulate the integrity and permeability of the BBB as well as immune and cancer cells infiltration via specific chemokine and cytokine network, angiotensin, apolipoprotein E (ApoE) and retinoic acid. Moreover, astrocytes influence the distribution of pericytes within NVUs [183]. One of the main astrocytic functions within NVU is the regulation of the content of water in the neuroparenchymal space, modulated via aquaporin 4 (AQP4), the main water channel protein [185]. Aquaporins are the proteins responsible for water transport in the brain and its movements between the four tissue compartments of CNS: intracellular, interstitial, vascular and ventricular [194,195]. They also play a role in cerebral volume regulation and formation of brain edema [196]. BBB functional integrity and low permeability of brain capillaries not only regulate the transport of water, ions and small molecules between various CNS compartments, but have also an impact on the absorption of various therapeutic agents from the blood, which may result in the restricted bioavailability and effectiveness of various drugs, which must reach malignant cells in adequate therapeutic concentration.

The functioning and organization of the BBB may be altered under certain pathological conditions, such as neuroinflammation or malignancy. Both primary CNS tumors, such as glioma, and metastatic are known to have a detrimental effect on the integrity and functioning of the BBB, resulting in changes of brain vasculature termed as the “blood–brain tumor barrier” (BTB). The extent of BBB damage is variable and depends on the type of tumor and its stage [197]. While in LGG tumors the changes in BBB are relatively small and BTB resembles BBB functioning, in more advanced and malignant high-grade gliomas BTB becomes disrupted and leaky. BBB alterations are most prominent in glioblastoma multiforme WHO IV grade [198]. The integrity of NVU in HGG tumors is disrupted due to displacement of astrocytes and pericytes, neurovascular decoupling, as well as altered pericyte populations [183]. Moreover, changes in endothelial TJs and transcytosis mechanisms also result in compromised permeability of endothelium in BTB. In addition, described above hypoxia, neoangiogenesis and tumor vessel co-option can also influence the NVU. The endothelial barrier properties may be compromised by the two main angiogenic pathways: VEGF and Ang-Tie2 signaling [199,200].

The permeability of blood vessels within the BTB shows a certain heterogeneity to circulating drugs, resulting also in uneven distribution of injected compounds within the tumor lesion. This depends on the type of tumor, primary or secondary, its histologic type, expression of various receptors, hydrophobic or hydrophilic features of the given compound and the degree of the cellular components of NVU disruption in the brain.

### 6.6. Brain-Tumor-Related Edema

The alterations of normal BBB function in gliomas, such as structural changes in the form of BTB, its increased permeability and reduced barrier functions of the CNS endothelium may result in brain tumor-related edema (BTRE). The typical, vasogenic mechanism of BTRE is related to the disruption of the BBB, in particular disturbed TJs expression and functioning, as well as the enhanced endothelial fenestrations [201], which allow leakage of fluids from the blood into the brain parenchyma [202]. In the formation of edema, VEGF-induced dysfunction of TJ proteins may play an important role [203].

Associated with intracranial tumors, BTRE is the abnormal accumulation of water inside the brain parenchyma and the leakage of plasma through dysfunctional cerebral capillaries [197,204]. In advanced cases, daily accumulation of these fluids may reach even 90 ml [205]. The stiffness of the skull and the accumulation of fluids inside this rigid, limited volume result in edematic swelling of the brain, the dramatic increase of the intracranial pressure (ICP) and lead to the brain herniation, and death [206], even in 60% of patients with GBM [207]. BTRE is the commonly observed symptom in GBM patients and one of the main causes of their morbidity and mortality [208]. BTRE may be observed in contrast-enhanced MRI, where lesions corresponding to tumor mass are surrounded by areas of peripheral heterogeneous enhancement of contrast, representing the disruption of the blood-brain barrier and vasogenic edema [208].

Cytotoxic edema, implicated in peritumoral swelling, is also an important mechanism leading to the formation of BTRE [205]. This type of glioma-induced edema is associated with neuronal cell death and neurodegeneration, which lead to further brain swelling and neurological deficits [209]. It was postulated that on the cellular level, the loss or reduction of astroglial polarity may be one of the main reasons for BTRE [185]. Polarity of astroglia is related with the specific accumulation of potassium and water channels, such as AQP4, in the superficial and perivascular astroglial endfeet membranes. Redistribution of AQP4 and a compromised directionality of water transport out of the cell may lead to cytotoxic edema [185].

### 6.7. Specific Role of Chemokines in the Neoangiogenesis of Glioma

Chemokines participate in many steps of glioma development, starting from tumor onset, and influence its proliferation, growth enhancement, aggressiveness, and influence various pathophysiological mechanisms [73]. It was shown that chemokines, especially from CXC and CC subfamilies, are also actively involved in tumoral angiogenesis, necessary for further development, progression, and spreading of glioma [210,211].

#### 6.7.1. CXCL8 and its Receptor CXCR2

CXCL8 is one of the most important chemokines in glioma and major regulator of this type of tumors pathogenesis. This chemokine belongs to the proangiogenic CXC-ELR (+) subfamily and is considered to be one of the most crucial chemokines for tumor vessel development, involved in tumor angiogenesis and survival of endothelial cells. In human gliomas, this chemokine is expressed and secreted at high levels both in vitro and in vivo [212]. Moreover, it was suggested that CXCL8 produced by malignant cells is critical to glial tumor progression and neovascularity. This chemokine exerts pleiotropic effects through two G-protein-coupled chemokine receptors CXCR1 and CXCR2 in the tumor microenvironment [212].

The proangiogenic activity of CXCL8 is predominantly mediated through the receptor CXCR2, which may also bind all other CXC ELR (+) chemokines. In the microenvironment of GBM, signaling of CXCR2 receptor is strongly activated and is responsible for tumor neovascularization [213]. High-affinity interactions of CXCL8 with endothelial CXCR2 trigger cell proliferation [212], resulting in consecutive migration of endothelial cells, which form a basic vessel structure with the central lumen [214]. CXCL8-CXCR1/2 axis mediates also the activation of MMPs in tumors, which participate in neoangiogenesis by their capability of degrading the basement membrane and ECM [215]. Nevertheless, CXCR1 may also contribute to proangiogenic functions of this chemokine, through independent small-GTPase activity [212].

Moreover, human HGGs resistant to antiangiogenic therapy express highly upregulated CXCL8-CXCR2 axis in tumor cells [172]. It has been suggested that this may be related to VM, the alternative mechanism for glioblastoma to form new blood vessels as described above, in which CXCR2 may play a significant role [172]. Importantly, in animal models of orthotopic GBM tumors the enhancement of VM was observed after antiangiogenic treatment with anti-VEGFR2 and anti-VEGF therapy. This alternative and independent on normal angiogenesis mechanism of the formation of new vessels is observed especially in GBM tumors expressing chemokine receptor CXCR2. Therefore, these tumors may exhibit a therapeutic resistance to conventional antiangiogenic therapies [216].

#### 6.7.2. CXCL12 and CXCR4 Receptor

Another chemokine that plays a crucial role in GBM neoangiogenesis is CXCL12, of which the functions are mediated through the receptor CXCR4 [217]. Both CXCL12 and CXCR4 are normally expressed in the brain, playing important roles in the CNS development. However, these proteins may act also as potent regulators of glioma development, promoting tumor growth and proliferation of glioblastoma stem cells through the activation of ERK1/2 and AKT pathways [218]. The contribution of CXCL12 and its receptor CXCR4 in glioblastoma may be considered as an example of tumor cells “hijacking” of physiological processes in CNS [219].

Although CXCL12 belongs to ELR (−) CXC subfamily, this chemokine exerts proangiogenic activity, such as stimulation of angiogenesis and inhibition of apoptosis [220]. These actions may be mediated by direct binding CXCR4 expressed by tumor vessels, or by promoting the recruitment of leukocytes [221,222]. CXCL12 gene expression in tumor cells is controlled by HIF-1α in direct proportion to reduced oxygen level in ischemic endothelial cells [220]. Hypoxic conditions, often present in brain tumors, induce HIF-1α expression, which further stimulates CXCL12 expression in tumor cells [223].

Moreover, it was shown that CXCL12-CXCR4 axis may promote VEGF production by glioma stem cells and mediate tumor angiogenesis via PI3K/AKT signaling [210]. Influenced by hypoxia, CXCL12 and CXCR4 cooperate and generate an amplification loop, where CXCL12 upregulate VEGF-A synthesis, which, in turn, further enhances the expression of CXCR4 [224]. It was revealed that CXCL12-CXCR4 axis is also involved in the angiogenesis in glioblastoma via HIF-1 and VEGF-dependent mechanisms [223]. Co-expression of HIF-1α, which plays a critical role in GBMs progression, and CXCR4 was observed in hypoxic regions of tumor, i.e., pseudopalisading glioma cells. Angiogenic tumor vessels were also strongly positive for CXCR4. Moreover, exposure to hypoxia produced a significant expression of CXCR4 and HIF-1*α* in glioma cells, while VEGF stimulated angiogenic response of CXCR4 in normal endothelial cells from brain microvessels [223].

The angiogenic switch of CNS tumors, which allows for recurrence of glioma, its quick progression and malignant transformation toward higher grades, may be also associated with proangiogenic CXCL12 activity. It was suggested that some changes in the expression of angiogenesis-related genes or proteins must emerge between an initial diagnosis of GBM and its recurrence after the treatment [225]. These changes of expression included VEGF-A and its receptors, VEGFR2 and VEGFR1, HIF1α, as well as CXCL12 and CXCR4, and were consistent between RNA and protein expression. It was assessed that at GBM recurrence after chemo-radiation, the expressions of CXCR4 and CXCL12 increased, while expressions of HIF1α and VEGFR2 decreased in comparison with their initial levels. These results confirm that the recurrence of glioblastoma is associated with a specific switch of the pattern of angiogenic factors expression, from VEGFR2-HIF1α to CXCL12-CXCR4 pathway, i.e., angiogenic switch to proangiogenic and protumoral CXCL12-CXCR4 signaling [225].

#### 6.7.3. CXCL16

CXCL16 is expressed constitutively in normal brain as a transmembrane multidomain molecule [226]. In normal conditions its expression is low and generally restricted to brain dendritic cells and vascular endothelial cells [226]. CXCL16 drives microglia toward an anti-inflammatory phenotype and plays a neuroprotective role against ischemia in normal brain [227]. The sole receptor for CXCL16, CXCR6 is widely expressed in the brain, including microglia, acting as endogenous protective factor against excitotoxic neuronal damage [228]. Moreover, CXCL16 also exists as a soluble molecule, inducing chemotaxis of CXCR6-expressing lymphocytes [229].

On the contrary, human glioma cells express CXCL16 chemokine abundantly, both on mRNA and protein level, which are further upregulated by TNFα and IFNγ. High amounts of CXCL16 are continuously released from glial cells [226]. In activated astroglial and glioma cells, transmembrane CXCL16 is shed to a soluble form by proteolytic cleavage, involving the activity of cell-surface MMPs [192]. CXCR6 is expressed only on a small subpopulation of tumor cells [230].

CXCL16 may be also an important factor in the modulation of microglia cell activity and their phenotype in GBM. It was reported that CXCL16 released by glioma can drive the polarization of microglia cells, called “glioma-associated microglia/macrophages” toward an anti-inflammatory/protumor phenotype [227]. This activity of CXCL16 may contribute to in the progression of the tumor. Moreover, CXCL16 interacting with CXCR6, also acts as a potent angiogenic mediator in tumorigenesis, inducing angiogenesis in autocrine signaling pathway involving HIF-1α [231].

#### 6.7.4. CCL2

CCL2 is a monocyte chemoattractant implicated in macrophage recruitment in normal conditions. This inflammatory chemokine is also a regulator of T cells [232] that controls polarization of Th2 lymphocytes into a more immunosuppressive T regulatory phenotype in vitro and in mouse models [233]. CCL2 may promote the differentiation of macrophages towards protumoral alternatively activated M2-type [234,235]. Moreover, proangiogenic properties of CCL2 were also demonstrated. It was shown that endothelial cells may express CCR2, the receptor for CCL2, and demonstrate chemotaxis and tube formation in response to CCL2 in vitro [97]. In addition, CCL2 was able to induce endothelial cell migration in a dose-responsive manner, acting as a direct mediator of angiogenesis [96].

As a chemokine that is abundantly produced by some tumors, CCL2 can also contribute to the progression and angiogenesis of malignant tumors, including CNS tumors. CCL2 may induce angiogenesis indirectly, as a monocyte chemoattractant, which results in the monocytic infiltrates [236]. Proangiogenic CCL2 may also have an indirect influence on tumoral angiogenesis by attracting TAMs. TAMs further secrete other proangiogenic factors such as VEGF, PDGF, transforming TGF-β, CXCL8, as well as gelatinases MMP-2 and MMP-9 [237]. Moreover, TAMs play a proangiogenic role in the tumor and secrete another proangiogenic chemokine, CXCL8, which additionally contributes to the progression of glioma [238]. Interestingly, blockade of CCL2 function with a neutralizing antibody resulted in a reduction of the infiltration of microglia/macrophages and in prolonged survival in mice model of gliomas [239]. This indicates that the recruitment of TAMs is strictly related to CCL2 secretion during glioma tumorigenesis. Furthermore, GBMs are infiltrated by a large number of TAMs, which may promote tumor resistance to antiangiogenic treatment. TAMs, of which the differentiation and survival depend on chemokine CCL2, may induce the expression of proangiogenic factors such as VEGF [238]. It was demonstrated in a rat model of GBM that the inhibition of CCL2 led to the blockade of macrophage recruitment and inhibition of angiogenesis, resulting in decreased tumor volume in CCL2-expressing GBM tissues. CCL2 expression can increase the resistance to anti-angiogenic treatment, while the suppression of CCL2 may play an important role in increasing the efficacy of this type of therapy in GBM, by inhibiting the recruitment of CCL2-dependent macrophages [240].

### 6.8. Noncoding RNAs

Results of recent research suggest that noncoding RNAs (ncRNAs), such as miRNAs, circRNAs, and lncRNAs, may also play an important role in glioblastoma angiogenesis. Although at least 90% of the human genome is transcribed, only ~3% of the genome contains protein-coding genes [241]. The remaining part of the genome encodes mainly ncRNAs, which play regulatory roles in maintaining a constant level of protein expression in the cells and controlling gene expression. Regulatory ncRNAs may be divided according to their length: as small noncoding RNAs (sncRNA), if they have less than 200 bases in length, or long noncoding RNAs (lncRNAs), while they are longer than 200 nucleotides [242]. These ncRNAs can alter gene transcription by several mechanisms of epigenetic processes.

MicroRNAs (miRNAs) are short ncRNAs with a length of 19–23 nucleotides that play important roles as post-transcriptional gene-regulation and in RNA silencing, by pairing to the mRNAs of protein-coding genes [243]. It was shown that some miRNAs, termed angiomiRs, have important functions in angiogenesis. These angiomiRs target key angiogenesis molecular drivers, such as metalloproteinases, hypoxia inducible factor 1 (HIF1), cytokines, and growth factors, including VEGF [244]. One of miRNAs regulating glioma neoangiogenesis is miR-101, which inhibits cell proliferation, angiogenesis, invasion, and metastasis. By promoting apoptosis, miR-101 acts as a tumor suppressor. Moreover, miR-101 is involved in cellular migration and the ability of endothelial cells to form capillary-like structures in glioblastomas [245]. Another angiomiR involved in glioma blood vessel development is MiR-137, which inhibits proliferation and angiogenesis of human glioblastoma cells by targeting enhancer of zeste homolog 2 (EZH2), an enzyme participating in histone methylation and transcriptional repression [246]. It was revealed that downregulation of miR-137 observed in GBM cells is associated with a poor prognosis in GBM patients [246].

LncRNAs constitute the largest and a very heterogeneous group of non-coding RNAs. They are mainly polyadenylated transcripts longer than 2200 pairs of bases, usually transcribed by RNA polymerase II [247]. LncRNAs interact with specific epigenetic proteins by modulating their activity, altering its localization or changing their role [241]. It was demonstrated that some lncRNAs, such as H19, lnc-POU3F3, HULC, Xist, HOTAIR, SNHG15 and PVT1 are upregulated in angiogenesis of glioma [241]. Moreover, miRNAs and lncRNAs form a complex network of specific interactions, which regulate each other’s expression and plays an important role in the cell pathophysiological processes of glioma. An example of such an interaction between noncoding RNAs is lncRNA CCAT2 with miR-424. It was demonstrated that lncRNA CCAT2 may promote glioma progression, cell proliferation and endothelial angiogenesis by inducing the PI3K/AKT signaling pathway. Moreover, this long noncoding RNA shares a complementary sequence with miR-424. While lncRNA CCAT2 was overexpressed in glioma cells, together with VEGF-A, expression of miR-424 was observed at low levels [248]. In addition, knockdown of lncRNA CCAT2 inhibited endothelial angiogenesis in glioma [248]. Another long noncoding RNA with many diverse biological functions, including the regulation of cell proliferation, differentiation and metabolism is lncRNA H19. It was shown that this ncRNA may also promote glioma angiogenesis [249]. Knockdown of lncRNA H19 in glioma cell lines resulted in decreased tube formation assays, whereas H19 overexpression was associated with significantly increased number of branches in tube formation assays. These activities were mediated through miR-138/HIF-1α/VEGF axis [249].

Circular RNAs (circRNA) are another type of noncoding RNA, with the unique structure of a covalently closed continuous loop. These circRNAs are generally expressed at lower levels, but are more stable than their linear nc-RNA counterparts. It was revealed that circRNAs may accumulate during ageing [250]. Moreover, it was also demonstrated that a high number of circRNAs are upregulated during neurogenesis [251]. Their function remains unclear, although the regulatory effects on tumorigenesis and tumor development were suggested [252]. In contrast to neural tissues, circRNAs are often downregulated in cancer and in other diseases associated with a high rate of cell proliferation [253]. Circular RNAs may be involved in the regulation of glioma cell proliferation, migration, and invasion. They may be also involved in glioma angiogenesis via miRNA-526b-3p/MMP2 pathway and RNA-binding proteins [254,255]. Circ-ATXN1 were significantly upregulated in glioma-associated endothelial cells in comparison with astrocyte-associated endothelial cells (AECs), whereas knockdown of circ-ATXN1 significantly inhibited endothelial cell viability, migration and tube formation [255].

**Figure 1:**
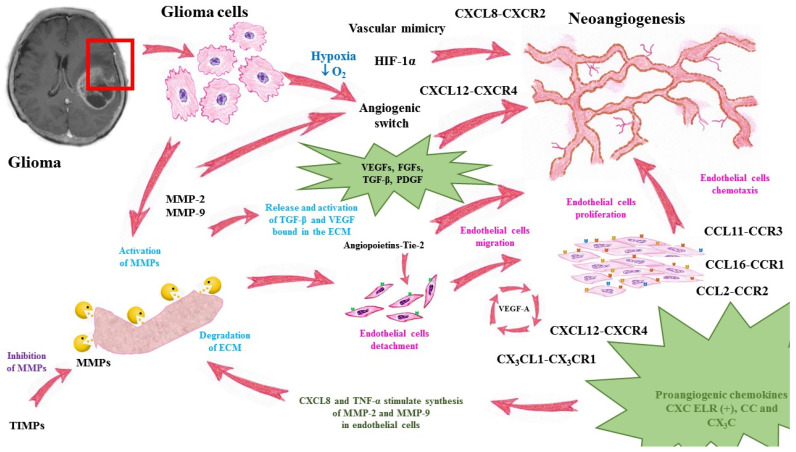
Mechanisms of glioma neoangiogenesis and functions of pro- and antiangiogenic factors. MMP—matrix metalloproteinase, TIMP—tissue inhibitor of matrix metalloproteinases, VEGF—vascular endothelial growth factor, TGF-β—transforming growth factor-beta, ECM—extracellular matrix, HIF-1α—hypoxia inducible factor alpha, TNF-α—tumor necrosis factor alpha, CXCL—chemokine C-X-C motif ligand, CXCR—CXC chemokine receptor, CCL—chemokine C-C motif ligand, CCR—CC chemokine receptor.

A summary of angiogenesis in glioma progression is presented in Figure 1.

## 7. Angiogenesis-Related Treatment Approaches and Implications for Novel Therapeutic Possibilities in Glioma

The current standard care for newly diagnosed GBM in adults includes maximal surgical resection of tumor, followed by combined radiotherapy and concomitant chemotherapy with temozolomide (TMZ), an alkylating agent [256]. Unfortunately, these conventional strategies for the treatment of glioma, accompanying surgery, i.e., chemotherapy and radiation, have poor specificity, dose sensitivity and bioavailability. In addition, further tumor progression and nearly universal mortality are experienced by majority of patients, resulting with a median survival shorter than 15 months. Therefore, certain supplementary therapies are needed, to improve efficacy of anti-glioma treatment.

Targeting tumoral angiogenesis is an example of such complementary approach, which aims to prevent the development of new blood vessels within glioma and normalize the tumor-associated vasculature. The fact that development of glioblastoma requires formation of new vessels and neoangiogenesis, which are mediated also by chemokines, has led to the conclusion that use of some inhibitory substances, which could stop the activity of selected chemokine-receptor axes, could be helpful as anticancer treatment in this group of malignant tumors. Blocking the proangiogenic activity of chemokines would, at least in some extent, limit tumor growth and reduce new vessels formation, which might support the current therapy. Therefore, some antiangiogenic therapies based on anti-VEGF activity, like the addition of bevacizumab to standard treatment with TMZ were suggested to improve patients’ survival [257,258].

One more antiangiogenic approach to glioma therapy is based on blocking the activity of some proangiogenic chemokines and/or their receptors. Such chemokine/chemokine receptor systems of particular interest in the regulation of antitumor mechanisms are CXC chemokines, especially CXCL8-CXCR2 and CXCL12-CXCR4 axes. Blocking of the CXCL8/CXCR2 alternative signaling pathway as an alternative therapeutic approach in HGG tumors was tested on mice glioma model [259]. Injections of SB225002, a CXCR2-antagonist, were performed after the implantation of tumor cells, which resulted in 50% reduction of tumor volumes, as verified using MRI assessment. Moreover, this treatment also led to the reduction of vessel density and accumulation of microglia/macrophages as well as enhanced interactions of these cells with tumor vessels, as it was shown in immunostaining. These results indicate that CXCR2-antagonist has a direct impact on proliferation of glioma and endothelial cells, inhibiting glioma growth, which represents a promising therapeutic target for glioma patients [259]. Another preclinical study on the same inhibitor of CXCR2 revealed that SB225002 treatment also resulted in a decreased tumor growth and incomplete vascular mimicry structures in the animal models of human orthotopic GBM [172]. Taken together, these results suggest that CXCR2 targeting can be combined with standard therapies of GBM to improve the therapeutic outcomes in clinical trials. Antiangiogenic therapy resistance in GBM patients may be also related to hypoxia-induced expression of CXCR4 receptor on tumor cells, microglial cells or glioma stem cells (GSCs). It was shown in an animal model of glioma that blocking this receptor with POL5551 inhibited CXCR4 binding to CXCL12 and reduced vascular density in tumors and glioma invasiveness, especially in combination with anti-VEGF antibody B20-4.1.1 [260]. These results suggest that adjunctive CXCR4 antagonists may be helpful in overcoming the resistance of antiangiogenic therapies in GBM patients.

However, the use of chemokine/chemokine receptor antagonists has been attempted only in a few clinical trials of cancer patients, either as a monotherapy, or in combination with immunotherapy. In particular, in GBM patients it is especially rare. Although some preclinical data suggest that this attitude might be beneficial in patients suffering from this type of tumors, to date these trials have primarily focused on other cancer types. Currently, there are some clinical trials directed at CXCR4 chemokine receptor (https://clinicaltrials.gov/ accessed on 16 May 2021) [261]. An ongoing clinical trial in GBM patients aims to evaluate the efficacy of CXCR4 antagonist Plerixafor (NCT03746080) [262]. Another trial is NCT02765165, evaluating a CXCR4 inhibitor, USL311 as a single-agent and in combination with Lomustine chemotherapy, in patients with relapsed/recurrent GBM (Phase 2) and in subjects with advanced solid tumors (Phase 1) [262].

## 8. Conclusions

Gliomas constitute a highly variegated group of malignant tumors of central nervous system. Their development and progression are strictly related with neoangiogenesis, which is controlled and regulated by numerous factors acting as stimulators or inhibitors of this complex process. Tumoral angiogenesis in glioma is characterized by certain mechanisms, which are not observed in physiological formation of normal new vessels. These mechanisms include hypoxia-induced angiogenic switch, which results from the alterations in angiogenic signaling pathways within glioma stem cells and allows the transition from low-grade to high-grade tumor. Aberrant blood vessel structure, with increased permeability, is typical for these tumors. Another characteristic feature of glioma angiogenesis is the formation of functional vessel-like networks, known as vascular mimicry. These neoangiogenic processes lead also to dysfunction of the blood–brain barrier and may result in tumor-related edematic swelling of the brain and dramatic increase of intracranial pressure.

Glioma angiogenesis is controlled and regulated by a broad spectrum of proangiogenic factors, which include members of VEGF family, FGF family, angiopoietins and angiopoietin-like proteins, cytokines from the TGF-β family, various enzymes from the MMPs family as well as proangiogenic CXC ELR (+) and CC chemokines and their receptors. Their signaling is involved in various aspects of glioma development and progression.

Therapeutic possibilities in glioma, especially glioblastoma multiforme, are of limited efficacy. The methods for treating these tumors used so far show little effectiveness, which results in very high mortality rate among patients, with an average survival time of about 1.5 years. This leads to the necessity of searching for new drugs. One proposed therapeutic strategy is antiangiogenic treatments, e.g., VEGF inhibitors. The new approach to therapy involves the use of substances that block the activity of proangiogenic chemokines and their receptors, although so far these trials have not gone beyond the phase of clinical trials. However, some biological conditions, such as the unique tumor and immune microenvironment interactions or the blood-brain barrier may represent additional substantial challenges in the development of novel glioma therapies. There is an urgent need for more effective therapeutic options, extensive efforts exploring immunotherapy and precision approaches in this devastating disease.

## Figures and Tables

**Table 1:** Proangiogenic factors in physiological angiogenesis.

Proangiogenic Factors	Mechanism of Action/Function	Author
VEGF-A	Proangiogenic growth factor	[21]
	Stimulation of mitogenesis in endothelial cells	[25]
	Stimulator of the migration of endothelial cells, monocytes/macrophages	[25,26]
	Increase of MMPs activity	[26]
FGF1 and -2	Promoters of physiological angiogenesis	[27,28,29]
	FGF-1/FGF-2: stimulation of endothelial cells proliferation, differentiation, and chemotaxis	[30]
	Induction of angiogenic response on blood vessels endothelium	[30,31]
	FGF-1: proliferation and differentiation of endothelial cells and smooth muscle cells for building arterial vessels	[30]
	FGF-2: endothelial cell proliferation and the physical organization of endothelial cells into tubelike structures	[31]
	Development of mature vessels and collateral arteries	[33]
	Degradation of ECM	[33]
	Upregulation of urokinase-type plasminogen activator (uPA) and MMPs in endothelial cells	[33]
Angiopoietins 1–4	Controlling microvascular permeability	[34]
	Endothelial cell migration and proliferation	[35]
	Vessel sprouting	[35]
	Ang-1: regulation of endothelial barrier function and stabilization of blood vessels	[35,38]
	Ang-1: regulation of vessel maturation, endothelial cell adhesion, migration, and survival	[35]
	Ang-2: blood vessel destabilization and disruption of angiogenesis	[39]
	Ang-2: inhibition of biding Ang-1 to Tie2	[39]
ANGPTLs 1–8	“Orphan” ligands—do not interact with Tie-1 and Tie-2 receptors	[41,42,43]
TGF-β family	TGF-β1: regulation of tissue morphogenesis, endothelial cell survival and tubular network formation	[44]
	TGF-β1: induction of vascular endothelial cells apoptosis related to VEGF/VEGFR-2 signaling	[46,55]
MMPs	Degradation of ECM proteins: proteoglycans, fibronectin, laminin	[56,62]
	Gelatinases MMP-2 and MMP-9: degradation of type IV collagen	[62,64]
	Disruption of tight junctions between pericytes and endothelial cells, formation of tunnels for new vessels, vessel sprouting	[62,65]
	Cleaving proangiogenic factors from ECM. Release and activation of proangiogenic TGF-β and VEGF bound in the ECM	[68,70]
CXC chemokines	Induction of angiogenesis	[84]
	Recruitment of proangiogenic hematopoietic cells and endothelial progenitors	[92]
	CXCL8: Main proangiogenic CXC chemokine, promotes endothelial cell migration, invasion, and proliferation	[89]
CC chemokines	CCL2: Main proangiogenic CC chemokine, chemoattractant for endothelial cells, formation of endothelial tube	[96]
	Receptor CCR2: expressed on endothelial cells.	[97]
	Regulation of MT1-MMP expression, clustering, and activity in endothelial cells	[98]
	CCL11: Induction of endothelial cells chemotaxis and proliferation	[93,99]
	Microvessel sprouting	[99]
	CCL16: Activation of angiogenic program in endothelial cells via CCR1, induction of endothelial cells migration	[94]
CX_3_C chemokine	CX_3_CL1: Induction of proliferation, migration, and formation of endothelial tube, stimulation of the angiogenesis	[100]
	CX_3_CL1 and CX_3_CR1 expressed on endothelial cells	[100,101]
	Microvessel budding, maturation, and vascular structural integrity	[103]

**Table 2:** Endogenous inhibitors of physiological angiogenesis.

Antiangiogenic Factor	Mechanism of Action/Functions	Author
Angiostatin	Induction of endothelial cells apoptosis	[106]
	Inhibition of endothelial cells proliferation	[107]
	Inhibition of MMPs-dependent endothelial cell migration	[108]
	Reduction of ERK-1 and -2 kinases phosphorylation	[107]
Endostatin	Downregulation of 12% genes of cell cycle control and apoptosis in endothelial cells	[110]
	Antimigratory effect in proliferating microvascular endothelial cells	[109,110]
	Interference with the proangiogenic activity of growth factors	[111]
	Inhibition of intercellular adhesion and between cells and ECM	[112]
VEGI	Signaling through death receptor 3 (DR-3) AND decoy receptor 3 (DcR-3)	[113]
	Autocrine proapoptotic factor in endothelial cells, antiproliferatory effect on endothelial cells	[114]
VEGFR-1/FLT-1	Inhibition of angiogenesis as a decoy receptor for VEGF	[117]
	Sequestration and trapping of VEGF, inhibition of VEGFR-2 activity	[117,118]
Neuropilin (NRP1)	Membrane-bound coreceptor and decoy receptor for VEGF165 isoform	[119]
	Modulation of VEGF binding and bioactivity, regulation of VEGF-induced angiogenesis	[119,122]
	Anti-VEGF activity of sNRP1	[123]
Antiangiogenic CXC ELR (−) chemokines	Angiostatic activity mediated mainly through CXCR3 receptor, which prevents the formation of endothelial tube	[125,126]
	Induction of newly formed cords regression in vitro and loss of blood vessels in vivo	[86]
	Inhibition of angiogenesis through a positive feedback loop, CXC ELR (−) chemokines stimulate the recruitment of NK and Th1 cells.	[127]
	CXCL10: dissociation of newly formed vessels, regression of blood vessels during wound healing, induction of endothelial cell death	[128]

## Data Availability

Not applicable.

## Abbreviations

AA	amino-acid
aFGF	acidic fibroblast growth factor
ALK1-7	activin receptor-like kinases
Ang	angiopoietin
ANGPTL	angiopoietin-like protein
ApoE	apolipoprotein E
AQP4	aquaporin 4
BBB	blood–brain barrier
BTB	blood–brain tumor barrier
BTRE	brain tumor-related edema
bFGF	basic fibroblast growth factor
BM	bone marrow
BMDCs	bone-marrow-derived cells
BMP	bone morphogenetic protein
CNS	central nervous system
CXCL	chemokine C-X-C motif ligand
CXCR	CXC chemokine receptor
CCL	chemokine C-C motif ligand
CCR	CC chemokine receptor
DcR-3	decoy receptor 3
DR-3	death receptor 3
EC	endothelial cells
ECM	extracellular matrix
EGF	epidermal growth factor
eNOS	endothelial nitric oxide synthase
ERK	extracellular-signal-regulated kinase
FGF	fibroblast growth factor
GBM	glioblastoma multiforme
HGF	hepatocyte growth factor
HGG	high-grade gliomas
HIF-1	hypoxia-inducible factor 1
IDH	isocitrate dehydrogenase
IFN	interferon
IL	interleukin
IL-12	interleukin-12
IMG	intussusceptive microvascular growth
ICP	intracranial pressure
ITSs	interstitial/intervascular tissue structures
KDR	kinase domain receptor
LGG	low-grade gliomas
LOH	loss of heterozygosity
LPS	lipopolysaccharides
MIP-1	monocyte chemoattractant protein-1
MMPs	matrix metalloproteinases
MT-MMP	membrane-type matrix metalloproteinase
NK	natural killer
NO	nitric oxide
NRP1	neuropilin
NVU	neurovascular units
PDGF	platelet-derived growth factor
PlGF	placental growth factor
PrGBM	primary GBM
SARS-CoV-2	severe acute respiratory syndrome coronavirus 2
ScGBM	secondary GBM
sNRP1	soluble form of neuropilin
TAMs	tumor infiltrating macrophages
TEK	endothelial-specific receptor tyrosine kinase
TGF-α	transforming growth factor alpha
TGF-β	transforming growth factor-beta
Tie-1	tyrosine kinase receptors with immunoglobulin-like and EGF-like domain 1
TIMP	tissue inhibitor of metalloproteinases
TL1A	TNF-like ligand 1A
TMZ	temozolomide
TNFSF15	TNF superfamily member 15
TNF-α	tumor necrosis factor alpha
TβRIII	type III TGF-β co-receptor
uPA	urokinase-type plasminogen activator
VEGF	vascular endothelial growth factor
VEGFR-1	receptor 1 for VEGF
VEGFR-2	VEGF receptor-2
VEGI	vascular endothelial growth inhibitor
VM	vascular mimicry
vSMCs	vascular smooth muscle cells
WHO	World Health Organization
